# Anxiety, not regulation tendency, predicts how individuals regulate in the laboratory: An exploratory comparison of self-report and psychophysiology

**DOI:** 10.1371/journal.pone.0247246

**Published:** 2021-03-12

**Authors:** Daisy A. Burr, Rachel G. Pizzie, David J. M. Kraemer

**Affiliations:** 1 Department of Psychology and Neuroscience, Duke University, Durham, NC, United States of America; 2 Program in Educational Neuroscience, Gallaudet University, Washington, D.C., United States of America; 3 Department of Education and Department of Psychological and Brain Sciences, Dartmouth College, Hanover, NH, United States of America; University of Colorado Denver, UNITED STATES

## Abstract

Anxiety influences how individuals experience and regulate emotions in a variety of ways. For example, individuals with lower anxiety tend to cognitively reframe (reappraise) negative emotion and those with higher anxiety tend to suppress negative emotion. Research has also investigated these individual differences with psychophysiology. These lines of research assume coherence between how individuals regulate outside the laboratory, typically measured with self-report, and how they regulate during an experiment. Indeed, performance during experiments is interpreted as an indication of future behavior outside the laboratory, yet this relationship is seldom directly explored. To address this gap, we computed psychophysiological profiles of uninstructed (natural) regulation in the laboratory and explored the coherence between these profiles and a) self-reported anxiety and b) self-reported regulation tendency. Participants viewed negative images and were instructed to reappraise, suppress or naturally engage. Electrodermal and facial electromyography signals were recorded to compute a multivariate psychophysiological profile of regulation. Participants with lower anxiety exhibited similar profiles when naturally regulating and following instructions to reappraise, suggesting they naturally reappraised more. Participants with higher anxiety exhibited similar profiles when naturally regulating and following instructions to suppress, suggesting they naturally suppressed more. However, there was no association between self-reported reappraisal or suppression tendency and psychophysiology. These exploratory results indicate that anxiety, but not regulation tendency, predicts how individuals regulate emotion in the laboratory. These findings suggest that how individuals report regulating in the real world does not map on to how they regulate in the laboratory. Taken together, this underscores the importance of developing emotion-regulation interventions and paradigms that more closely align to and predict real-world outcomes.

## Introduction

Stressful experiences are an inevitable aspect of life. Although most individuals can commiserate about shared distressing events, such as a traffic jam or a breakup, there is significant variability in how individuals respond to stressors [1]. Consider two individuals, Kathleen and Tom, who may both encounter the same negative experience, such as failing an exam, but Kathleen may reframe the failing grade as a consequence of not studying enough after starting to feel upset and embarrassed, motivating her to hit the books. In contrast, Tom may be very anxious and mask his emotions instead of actively processing the experience. Kathleen and Tom naturally chose to respond to the stressor using different strategies and consequently experienced it in different ways.

Various strategies exist for trying to change the intensity, duration, and quality of emotional experiences, collectively referred to as emotion regulation. Two common strategies for trying to modulate the emotional impact of a stimulus are cognitive reappraisal (henceforth reappraisal) and expressive suppression (henceforth suppression; Gross, 2008 [2]). Reappraisal, as exemplified by Kathleen, is characterized by actively reframing a stimulus in order to change its meaning or value [3–5]. Multiple studies have illustrated that reappraisal helps reduce negative affect and combat the potentially harmful effects of chronic stress [3, 6–9]. Suppression, as exemplified by Tom, is considered an avoidance-based emotion regulation strategy that aims to modulate the response to an emotional stimulus by reducing the outward expression of negative emotion [2, 5]. However, suppression often fails to durably alter the associated internal experience [10]. Reappraisal and suppression also differ in *when* they occur in the lifecycle of an emotion; reappraisal typically occurs earlier in the emotional experience and is considered an antecedent-focused strategy, whereas suppression typically occurs later in the emotional experience and is considered a response-focused strategy [2, 5, 11, 12].

Research has explored how the tendency to reappraise and suppress relates to larger health outcomes. Specifically, previous research has demonstrated the myriad ways that anxiety influences how individuals process and regulate emotions. For example, individuals who tend to suppress experience heightened anxiety, whereas those who tend to reappraise report decreased anxiety [6, 9, 13–17]. In addition to anxiety influencing how individuals tend to regulate their emotions, it also impacts how they respond to regulation instructions/interventions [18] and process negative stimuli in general [19].

Psychophysiology offers an additional lens through which to measure anxiety and emotion regulation. This line of research has shown that suppression leads to reduced activity in the corrugator muscle—a muscle important for frowning and implicated in feeling anger and sadness [20–22]—and the levator muscle—a muscle associated with the expression of disgust [23–26]. Despite suppression resulting in a decrease in the outward expression of negative emotion, individuals typically exhibit increased sympathetic arousal and skin conductance, usually measured with electrodermal activity (EDA; [15, 27–31]) These findings suggest that suppression may have a counterintuitive effect that modulates the outward expression of emotion, but fails to durably address the underlying emotional experience [10]. On the contrary, reappraisal has been shown to decrease both the outward expression of negative emotion *and* the internal subjective experience [32]. Taken together, this suggests that suppression leads to a lack of coherence between subjective and psychophysiological components of emotion [10, 33]. Individuals suffering from excessive anxiety tend to automatically exhibit these same response patterns. For example, individuals who suffer from excessive anxiety experience increased arousal and activity of frowning muscles in response to negative stimuli [34]. In fact, Botulinum toxin (colloquially referred to as Botox) therapy has been shown to relieve anxiety by reducing automatic excitation of frowning muscles [35].

### Current study

The vast majority of research on emotion regulation has focused on reappraisal and suppression. Moreover, the wealth of research on anxiety and emotion regulation emphasizes reappraisal and suppression [13–16]. Relatedly, the ERQ—the gold-standard scale of natural regulation tendency—measures reappraisal and suppression [36]. Therefore, the current study focused on these strategies and informed predictions based on existing research.

Research describing the psychophysiological correlates of anxiety and emotion regulation is typically conducted in the laboratory. Participants will follow instructions to regulate while viewing emotionally-evocative stimuli, as well as complete a series of self-report measures so researchers can gain insight into their subjective experience. However, it is unclear if participants behave similarly inside and outside the laboratory—how individuals regulate in an experiment may not align to how they regulate in the real world. To bridge this gap, affective self-report measures often attempt to capture how individuals tend to experience and regulate emotions in the real world. However, this assumes individuals are aware of and can accurately characterize their experience to answer questions such as “what did I do to feel less negative?” [37]. Importantly, this reflective and metacognitive skill may be particularly difficult for individuals with anxiety [38]. Individuals with anxiety often ruminate about the self and suffer from heightened self-consciousness, which is associated with deficits in perception [38]. Self-report is also limited by demand characteristics [39, 40]. Moreover, self-report measures are not always investigated for coherence with other affective measures, such as psychophysiology, which may be less affected by demand characteristics [41, 42]. To address these gaps, we computed multivariate psychophysiological profiles of uninstructed (natural) regulation in the laboratory and investigated the coherence between these profiles and a) self-reported anxiety and b) self-reported regulation tendency.

Instead of only measuring changes in facial musculature, typically measured with electromyography (EMG), or EDA in isolation, we combined these channels to more comprehensively index how individuals regulate in a variety of contexts. We first validated psychophysiological profiles of reappraisal and suppression and tested how these profiles vary based on trait anxiety. Based on this proof-of-concept model testing the psychophysiological signature of emotion regulation, we conducted an exploratory analysis to examine the coherence between self-reported and psychophysiological indices of emotion and emotion regulation.

Prior research has emphasized that emotions involve the coordination of subjective, behavioral and psychophysiological response systems [33]. Moreover, coherence among these response systems indicates higher levels of well-being [33]. However, research has yet to explore how indices of emotion regulation converge. We aimed to investigate how subjective self-reporting of anxiety and natural regulation tendency influenced how individuals spontaneously regulate emotion in the laboratory. To accomplish this goal, we computed a multivariate psychophysiological dissimilarity metric that captures how much participants naturally reappraised and suppressed, in the absence of regulation instructions. We then compared this metric with two common self-report measures of the subjective experience of emotion and emotion regulation—natural regulation tendency and trait anxiety.

Foundational research on the psychophysiological correlates of emotion regulation and anxiety guided predictions in the current study. Anxiety fundamentally influences, and even predicts, how individuals experience and regulate emotion [14]. Individuals with heightened anxiety tend to naturally suppress and exhibit increased psychophysiological arousal in response to negative emotion [13, 15, 16]. Therefore, we predicted that participants who tend to suppress negative emotion would exhibit higher skin conductance. Similarly, we predicted that these patterns would be particularly true for individuals with heightened anxiety, as they are more likely to naturally suppress negative emotion [13–16]. Based on the mechanisms of suppression [5, 8, 16, 43–45], we similarly predicted that increased suppression would be negatively correlated with EMG activity. To the extent that reappraisal effectively reduces negative affect and facial expressions change accordingly, we similarly predicted that increased reappraisal would be negatively correlated with EMG activity [32].

Based on the extent to which anxiety shapes perceptions and experiences of emotion [13, 14]; we collectively predicted that participants would regulate differently depending on how anxious they were. Specifically, based on prior research [13–17], we predicted that participants with higher anxiety would exhibit uninstructed (natural) psychophysiological profiles that resembled suppression and participants with lower anxiety would exhibit uninstructed (natural) psychophysiological profiles that resembled reappraisal. Similarly, we predicted that participants who reported frequently reappraising would exhibit uninstructed (natural) psychophysiological profiles that resembled reappraisal and participants who reported frequently suppressing would exhibit uninstructed (natural) psychophysiological profiles that resembled suppression.

## Materials and methods

### Participants

Fifty-eight undergraduate students were recruited to participate in this study from a pool of 488 students enrolled in introductory psychology and neuroscience courses in a small college in New England. Data collection was part of a different study and recruitment was therefore based on scores from the Math Anxiety Rating Scale [46], a self-report questionnaire that assesses anxiety and negative affect directed toward mathematics. All eligible participants from this different study were included in the present study. Six students were excluded from further analyses: one participant was excluded for extremely low accuracy (not significantly different from chance-level responding, ~50%) on the math and/or analogy trials (see [47]), two participants were excluded for having a large number of missing responses (> 3 standard deviations above the mean number of non-response trials), and three students were not included in data analysis because they did not complete the task due to fatigue or power failure. Fifty-two participants were included in the dataset for analysis (*M*_Age_ = 19.56, *SD*_Age_ = 1.14, 63.5% female). All participants provided written informed consent to participate in a psychophysiological experiment and all procedures were approved by the Dartmouth Committee for the Protection of Human Subjects. Participants were compensated with course extra credit or cash.

This sample size was idealized for a different empirical question (see [47]) and results should be interpreted cautiously. However, our sample size in line with or larger than numerous studies that use the same emotional stimuli and similarly investigate the effects of regulation strategies [3, 48].

### Training

Participants were told that they would see a cue at the beginning of each block of trials instructing them how to engage with the stimuli over the course of the following twenty trials. This cue would either explicitly instruct them how to regulate their emotions (“REAPPRAISE” or “SUPPRESS”) or direct them to engage with the stimuli as they naturally would (“LOOK”), constituting an uninstructed condition [49]. In the uninstructed condition, participants were not directed to refrain from regulating their emotions, but rather were directed to spontaneously engage, allowing them to regulate as they see fit.

Participants were trained on the two instructed emotion regulation strategies (“REAPPRAISE” and “SUPPRESS”). This twenty-minute training mirrored training used in published research on emotion regulation and has been established to teach participants how to reappraise and suppress [5, 8, 16, 43–45, 50]. Participants practiced using each instructed emotion regulation strategy and described the strategy in their own words. During the blocks of suppression trials (“SUPPRESS”), participants were instructed to monitor and control their facial expressions to maintain a neutral expression, such that if they experienced any emotion, no one would know what they were feeling. During the blocks of reappraisal trials (“REAPPRAISE”), participants were instructed to use a method of reinterpreting the meaning of the stimuli to feel less negative about it [6, 44]. Specifically, participants were instructed to use an emotional distancing reappraisal strategy and were told to imagine looking at the stimuli from an objective perspective. For example, participants could imagine that the image had a broader context that made it less negative by creating a personal narrative (e.g., “Although at first I thought he looked lonely and sad, I imagined the man pictured waiting at the window was waiting for his grandchildren who were playing outside”). Participants could also adopt a perspective that allowed them to focus on the technical details of the photograph in order to feel less negative, such as imagining that they were a photographer examining the picture or a medical professional evaluating pictures of individuals who had been injured. After being trained on reappraisal and suppression, participants described these strategies to the experimenter, and the experimenter provided feedback when necessary. Participants practiced problems in twelve categories of stimuli (three (emotion regulation strategy: look, reappraise, suppress) x four (stimuli: negative, neutral, analogy, math), and reported how they used the appropriate regulation strategy. The experimenter provided verbal feedback and redirected responses to align with the emotion regulation strategy instructions when necessary. The present study only discusses uninstructed, reappraise and suppress blocks while engaging with negative stimuli (twenty trials per condition = sixty trials per participants). To review the other stimuli conditions, see Pizzie & Kraemer, 2018 [47].

### Task

Participants were directed to apply the instructed emotion regulation strategies (“REAPPRAISE” or “SUPPRESS”) or engage naturally (“LOOK”) to four different types of stimuli: negative images, neutral images, math problems and analogies. Images were obtained from the International Affective Picture System [19] based on their high negative valence and arousal ratings (*M*_valence_ = 1.74, *SD*_valence_ = 0.17, *M*_arousal_ = 6.37, *SD*_arousal_ = 0.58). However, the present study only discusses uninstructed, reappraise and suppress blocks while engaging with negative stimuli (twenty trials per condition = sixty trials per participants). To review the other stimuli conditions, see Pizzie & Kraemer, 2018 [47].

**Fig 1:**
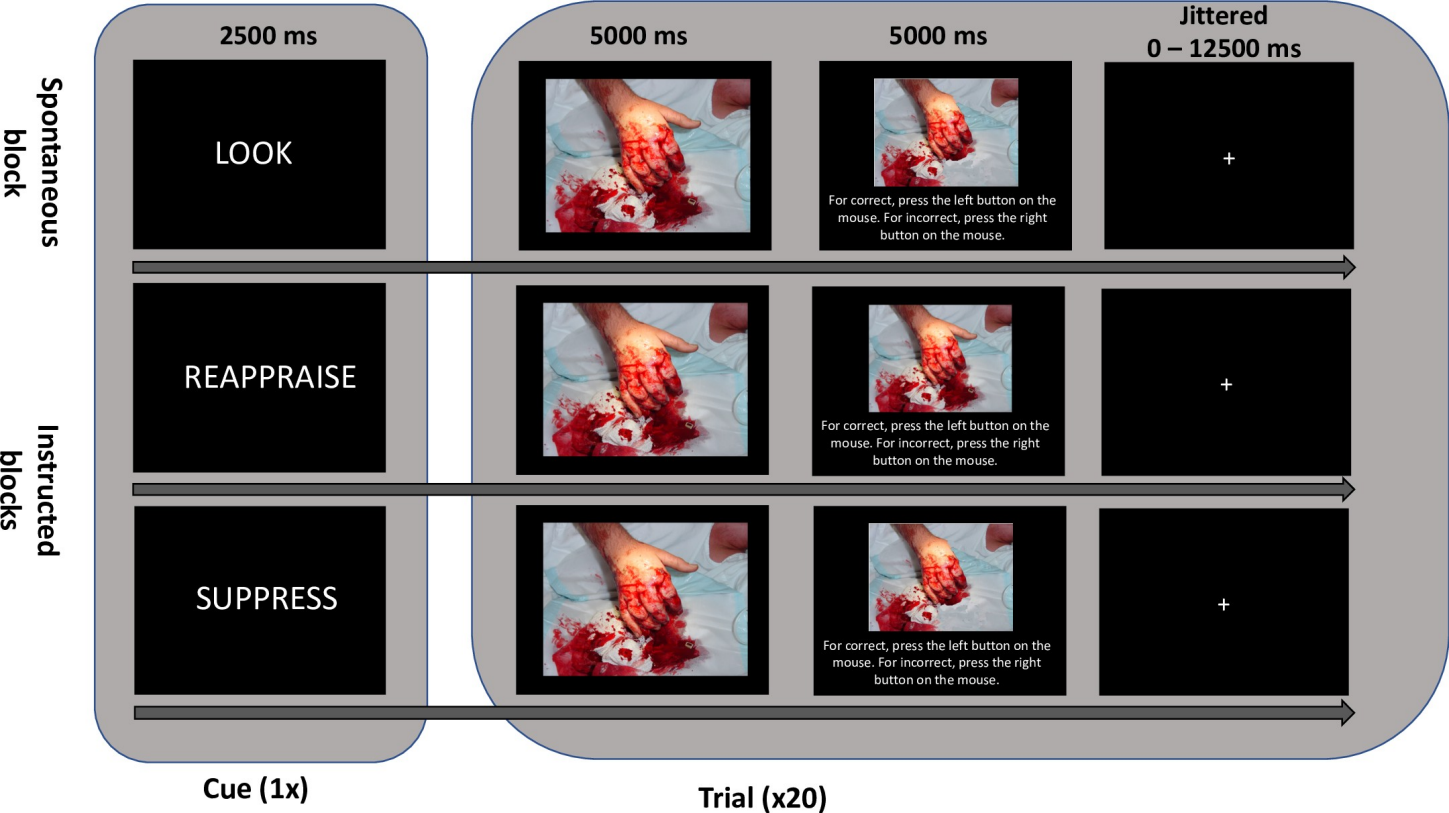
Trial cadence. Trial cadence for emotion regulation task depicting the instructed conditions (“REAPPRAISE” and “SUPPRESS” cues) and uninstructed condition (“LOOK” cue). Participants were given a cue at the beginning of a block of twenty trials. Participants first saw an original stimulus for 5000 ms. On the subsequent answer screen, participants either saw the identical image again, as depicted in the middle (REAPPRAISE) row, or a slightly altered image, as depicted in the top (LOOK) and bottom (SUPPRESS) rows. With a button press, participants indicated whether the image was identical (“correct”) or had been altered (“incorrect”). Stimuli were not repeated between conditions and were randomly presented within blocks. The order of emotion regulation conditions was randomized. Images from the International Affective Picture System were used in the experiment. However, the above image is not from this database. The photo used is credited to https://litfl.com/clinical-cases/ and was edited and reused here with permission under the Creative Commons Attribution-ShareAlike 3.0 Unported license (https://creativecommons.org/licenses/by/3.0/legalcode).

At the beginning of each block of trials (sixty trials), participants were presented with a cue directing them to use an emotion regulation strategy (Fig 1). Although participants can rapidly switch between regulation strategies from one trial to the next, this blocked strategy allowed us to make within-subject comparisons across regulation strategies, but reduced the amount of confusion or distraction that might be created by rapidly switching emotion regulation strategy on each trial [51]. The order of all blocks and trials were randomized. For each negative image trial, participants viewed an image for 5000 ms and were instructed to maintain attention on the image, then were presented with an answer screen with either an identical image or an image that had been slightly altered (i.e., subtle alterations made using photo editing software) for 5000 ms. Participants indicated with a button press whether they thought the answer image was identical to the first image or not. Accuracy was determined by whether the participant correctly indicated if the image that they observed had been altered or was exactly identical to the original image. Trials were separated by a jittered inter-trial-interval. Participants completed sixty negative image trials, with twenty trials in each emotion regulation condition. Blocks were presented in a randomized order and all participants completed twelve blocks of trials (three emotion regulation strategies x four stimulus categories). The present study only discusses psychophysiological data from the 5000 ms stimulus window during uninstructed, reappraise and suppress blocks while engaging with negative stimuli (twenty trials per condition = sixty trials per participants). To review the other stimuli conditions, see Pizzie & Kraemer, 2018 [47]. To review all data, see Pizzie & Kraemer, 2018 [47].

At the end of the experiment, participants completed a series of self-report questionnaires and provided demographic information. In these analyses, we focus on how trait anxious participants are, which was measured by self-reported trait anxiety (Spielberger State-Trait Anxiety Inventory—trait subscale, STAI; [52]). In addition to measuring uninstructed modulation of negative emotion in the “LOOK” condition, we measure natural regulation tendency with the Emotion Regulation Questionnaire (ERQ; [36]). The ERQ includes two subscales—reappraisal (e.g. “I control my emotions by changing the way I think about the situation I’m in”) and suppression (e.g., “I control my emotions by not expressing them”). Each facet is scored separately, resulting in independent reappraisal and suppression tendency scores.

### Psychophysiological data collection

We collected EDA and facial EMG data throughout the task. For each trial, we used responses from the 5000 ms stimulus window and the 5000 ms response window. In order to account for the shape of the biological functions that represent psychophysiological data (i.e., skin conductance response), we calculated the area under the curve (AUC) to separately model the responses during the 5000 ms stimulus window [53]. AUC is an established method for measuring mean and overall level of skin conductance over a period of time, more similar to measuring skin conductance level during the stimulus window. This method does not require that activity during intervals be categorized as specific skin-conductance responses or not [53]. To account for individual differences in baseline levels of psychophysiological reactivity, the AUC measurements were z-scored within each subject in order to mean-center the comparisons across conditions. Processing of psychophysiological data was done using standard procedures in BioPac’s AcqKnowledge software [54].

#### Electrodermal activity

We measured sympathetic nervous system activity with EDA from the hand of each participant by attaching a pre-gelled Ag/Ag-Cl electrode to the second phalanx of the index and middle finger on the non-dominant hand [55] The data were sampled at rate of 1,000 Hz and preprocessed by passing the signal through a band pass filter, isolating the signal between .5 Hz and 60 Hz [56]. The data were first processed with a BioPac amplifier, with a gain of 5 mΩ/V., and the signal was DC restored. The data were processed using BioPac’s AcqKnowledge software and mean value smoothed using a 500 ms window to amplify the signal-to-noise ratio.

#### Electromyography

We measured changes in electrical activity in facial musculature using EMG from the corrugator supercilii—a muscle group adjacent to the eyebrows frequently associated with increased negative affect [23, 24]—and levator labii superioris—a muscle group implicated in disgust reactions [25]. We recorded from the left side of the face using 4mm Ag/Ag-Cl electrodes that were filled with isotonic gel [20, 57]. We exfoliated the skin at each site with a gel to lightly abrade the skin and cleaned with an alcohol wipe before attaching the electrodes and checked that impedance at each site was at acceptable levels (< 10 Ω). We sampled EMG signals at 1,000 Hz and initially processed with a BioPac amplifier with a gain of 2000. We then processed EMG signals with a 100 Hz high pass filter and a 500 Hz low pass filter (effectively a band pass filter). Although EMG signals may begin at frequencies below 100 Hz (EMG frequency signal typically ranges between several Hz to 500 Hz; [55], we used a high pass filter in order to more conservatively filter the signal and eliminate 60 Hz electrical noise that may interfere with the signal. We demeaned and rectified the data to produce a positive signal and calculated AUC to model responses during the 5000 ms stimulus window [53]. AUC is an established method for measuring mean and overall level of changes in electrical activity in facial musculature over a period of time and does not categorize specific responses as a change in facial musculature or not [53].

### Data analysis

The present study only discusses uninstructed, reappraise and suppress blocks while engaging with negative stimuli. Stimuli not discussed were collected as part of a different study investigating a different empirical question (see [47]). Similarly, data were collected on fifty-eight participants who were recruited for this study investigating a different empirical question and analyses were conducted on fifty-two of those participants who passed standards of data quality (see Participants). Participants were recruited to obtain a sample and effect size for this different study (see [47]). Therefore, the current study is exploratory in nature and effects should be interpreted cautiously.

**Fig 2:**
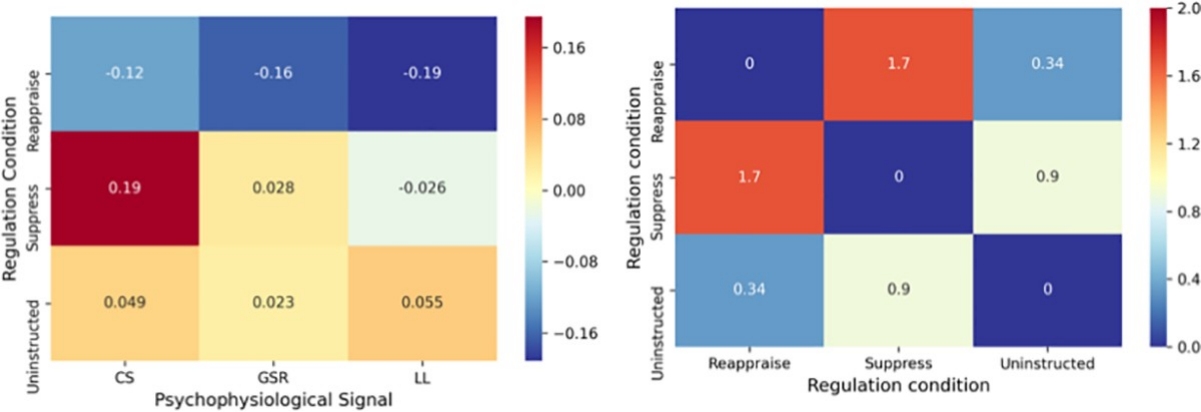
Sample patterns of psychophysiological activity and multivariate distance. Pattern of psychophysiological activity for each regulation condition for a single sample participant (left) and correlation distance among regulation conditions based on sample participant’s unique profile (right). CS = corrugator supercilii, GSR = galvanic skin response (skin conductance) and LL = levator labii. The value corresponding to the psychophysiological signal for each regulation condition (left) is the average activity for that signal across all twenty trials in that regulation condition and participant. Psychophysiological data are within-subject z-scored. Dissimilarity (right) is measured with correlation distance (ranging from 0 to 2, with 0 representing perfect correlation, 1 representing no correlation and 2 representing perfect anti-correlation). Smaller y-axis values represent greater similarity.

To quantify uninstructed regulation, we computed a continuous measure of multivariate psychophysiological dissimilarity among emotion regulation conditions. We computed a multivariate physiological profile of each participant’s emotion regulation conditions to measure dissimilarity across types of emotion regulation. To do this, we first computed each participant’s multivariate psychophysiology profile of regulation (Fig 2). Each participant has their own unique pattern of skin conductance, corrugator and levator activity when they are reappraising, suppressing and naturally engaging (uninstructed). The value corresponding to the psychophysiological signal for each regulation condition is the average activity for that psychophysiological signal across all twenty trials in that regulation condition. It was crucial to compare how participants naturally responded to negative stimuli versus how they responded when being instructed to suppress or reappraise. This approach allowed us to compute a multivariate psychophysiological profile of participants’ natural regulation style, and compare that to their suppression and reappraisal profiles to quantify the extent to which participants naturally suppressed and reappraised.

Based on these unique profiles, we computed the distance between each condition as a measure of how dissimilar their instructed and uninstructed profiles were (Fig 2). Each participant’s unique psychophysiology profile of each regulation condition was then compared using correlation distance as a measure of dissimilarity among conditions in order to quantify how much that participant was naturally reappraising (dissimilarity between uninstructed and instructed reappraisal) and suppressing (dissimilarity between uninstructed and instructed suppression). We used correlation distance to capture the relationship among regulation conditions in order to value the inherent relativity of participants’ psychophysiological signals when engaging with stimuli (ranging from 0 to 2, with 0 representing perfect correlation, 1 representing no correlation and 2 representing perfect anti-correlation; https://docs.scipy.org/doc/scipy-0.14.0/reference/generated/scipy.spatial.distance.correlation.html). Comparing multivariate psychophysiological distance is a well-validated technique when working with high-dimensional data [58, 59]. Here, we explore if this approach can be used to understand the relationship between how people naturally engage with versus follow instructions to regulate negative stimuli.

## Results

### Descriptives

**Fig 3:**
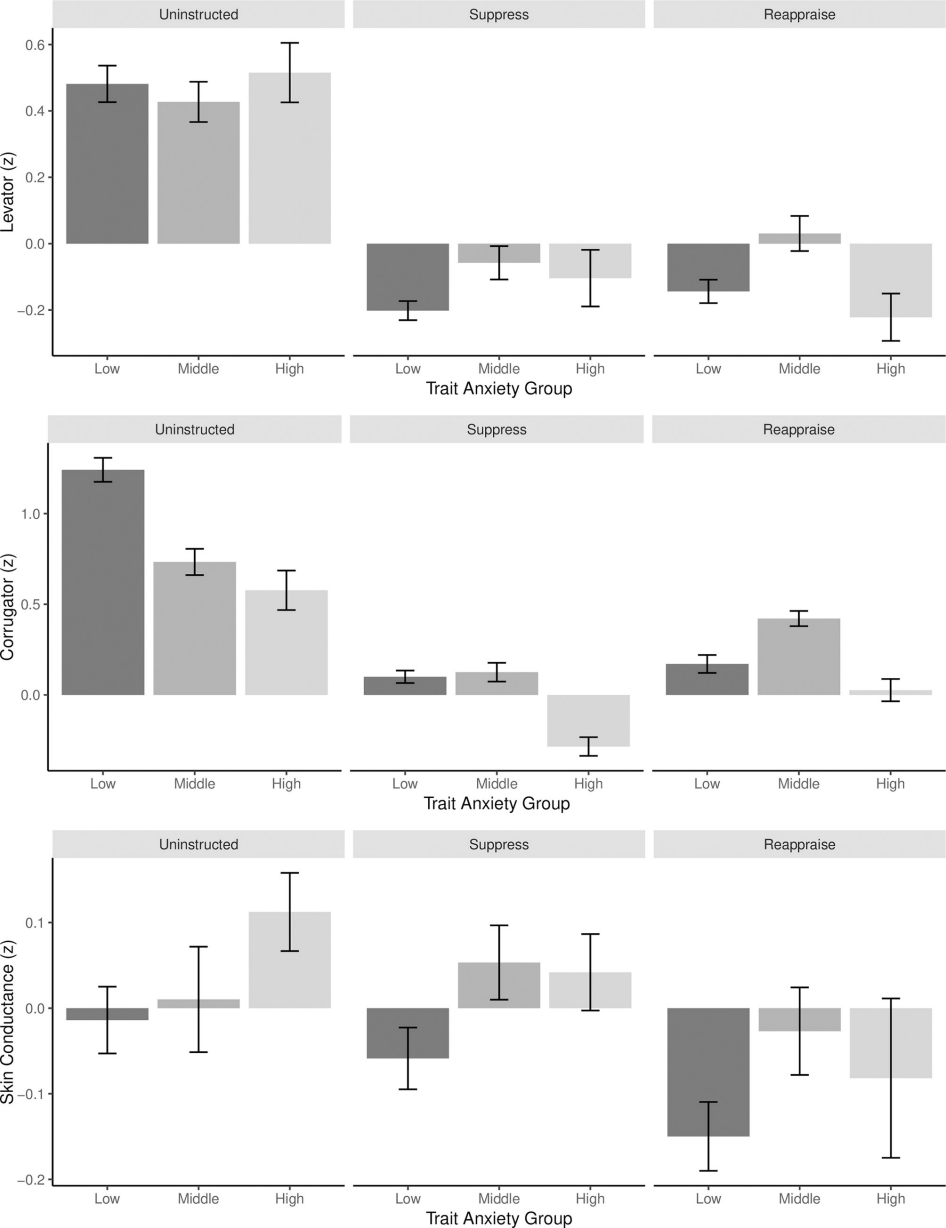
Psychophysiological activity by regulation condition and trait anxiety. Mean physiological activity for each measure in each emotion regulation condition, grouped by trait anxiety. Although trait anxiety (State-Trait Anxiety Inventory; STAI) is a continuous measure, for visualization purposes it is divided here into three groups corresponding to below one SD below the mean (Low), within one SD of the mean (Middle) and above one SD above the mean (High). All psychophysiological data are within-subject *z*-scored across all conditions in the study.

Before analyzing the psychophysiological correlates of emotion regulation, we explored how mean levels of skin conductance, corrugator and levator activity varied based on emotion regulation condition and level of trait anxiety (Fig 3). Although trait anxiety was a continuous measure, for illustrative purposes, it is divided into three groups corresponding to one SD below the mean (Low, *M*_z-scored trait anxiety_ = 1.63, *n* = 24), within one SD of the mean (Middle, *M*_z-scored trait anxiety_ = 2.22, *n* = 19) and one SD above the mean (High, *M*_z-scored trait anxiety_ = 2.86, *n* = 9).

### Can psychophysiological activity differentiate reappraisal and suppression?

Before comparing psychophysiological and self-reported indices of emotion and emotion regulation, we first tested if there was a pattern of psychophysiological activity that reflected reappraisal and suppression. Based on prior research demonstrating that trait anxiety impacts psychophysiological reactivity to negative stimuli, we also tested how this pattern differed based on level of STAI-trait subscale. In order to test this, we determined the psychophysiological correlates of reappraisal and suppression in our sample with a mixed-effects logistic regression that allowed for random participant intercepts. This model tested if anxiety and activity from all three psychophysiological signals—skin conductance, corrugator EMG, and levator EMG—differentiated the reappraise versus suppress conditions. In other words, we modeled which patterns of anxiety, skin conductance and EMG activity reflected reappraisal versus suppression.

**Fig 4:**
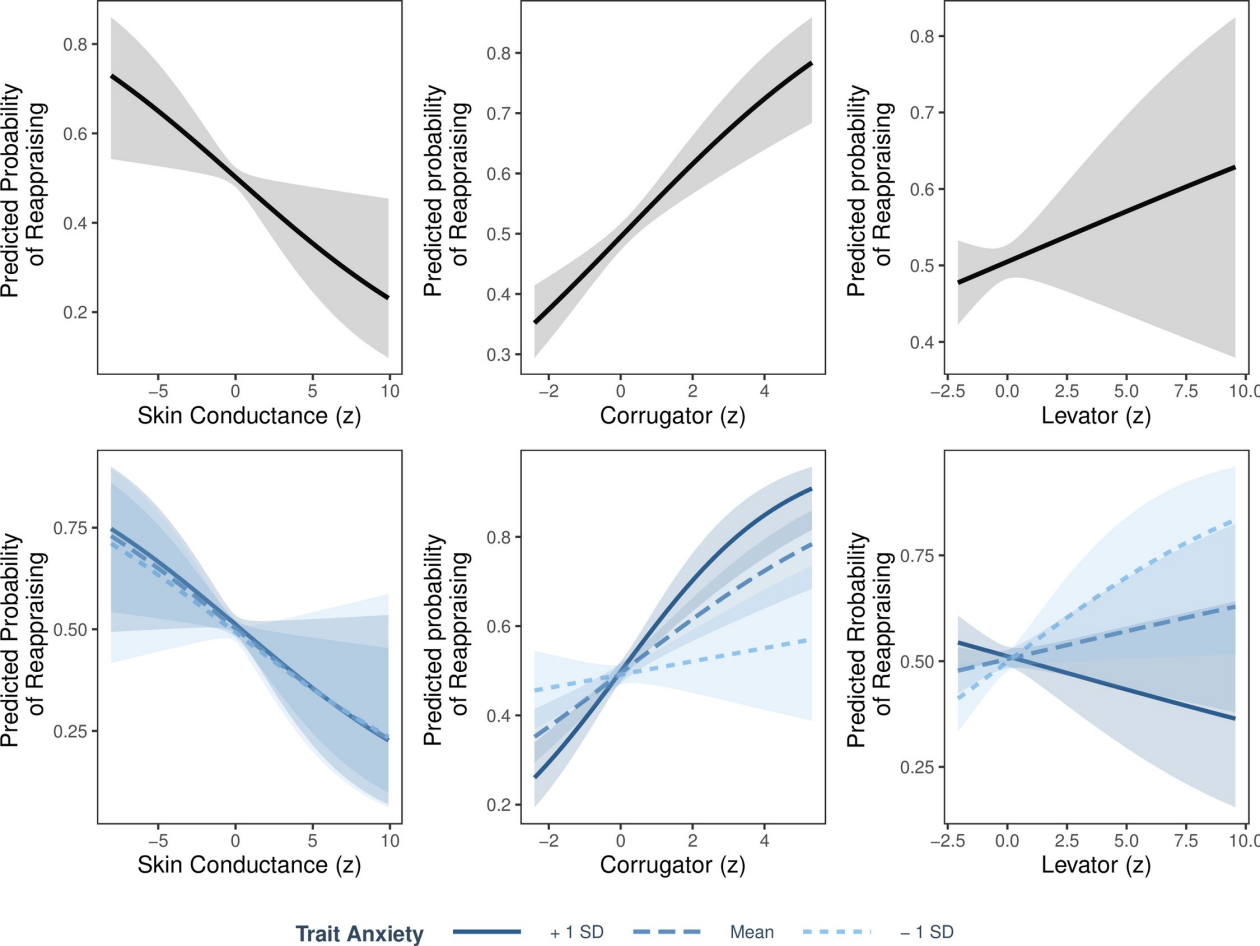
Psychophysiological patterns associated with reappraisal versus suppression. Logit function from a mixed-effects logistic regression predicting if participants were in the reappraise or suppress condition. Top: significant effect of skin conductance (left), significant effect of corrugator (middle) and non-significant effect of levator activity (right) on the predicted probability of being in the reappraisal condition as opposed to the suppress condition. Bottom: non-significant interaction between skin conductance and trait anxiety (left), significant interaction between corrugator and trait anxiety (middle) and significant interaction between levator and trait anxiety (right) on the predicted probability of being in the reappraisal condition as opposed to the suppress condition. All psychophysiological data are within-subject *z*-scored.

Fig 4 illustrates the results of this logistic regression, which predicted how likely participants were in the reappraise as opposed to suppress condition. In line with prior research [15, 27] and the descriptive differences between reappraise and suppress conditions observed in Fig 3, participants with increased skin conductance were less likely to be reappraising and more likely to be suppressing (β = -.12, 95% CI [.-.22 -.02], *p* = - .02). Similarly, participants exhibiting decreased corrugator activity were less likely to be reappraising (β = .24, 95% CI [.15, .34], *p* < .001) and this relationship was strongest for those with higher anxiety (β = .36, 95% CI [.15, .58], *p* < .001). In addition, participants exhibiting decreased levator activity and who had higher anxiety were less likely to be reappraising and more likely to be suppressing (β = -.23, 95% CI [-.43, -.04], *p* = .02). Levator activity (β = .06, 95% CI [-.05, .16], *p* = .29) and the interaction between skin conductance activity and trait anxiety (β = -.01, 95% CI [-.22, .2], *p* = .89) were non-significant predictors of instructed regulation condition.

### Do subjective and psychophysiological measures of emotion and emotion regulation align?

We then used this signature of emotion regulation as a comparison against uninstructed (“LOOK”) condition to understand how much participants were naturally suppressing and reappraising. By computing the multivariate psychophysiological dissimilarity among regulation conditions, we quantified how close participants’ uninstructed and instructed (reappraise and suppress) conditions were (i.e. how similar or dissimilar their multivariate psychophysiological profiles were). In other words, the multivariate psychophysiological dissimilarity between each participant’s unique uninstructed and reappraise conditions represents how much they naturally reappraise in the uninstructed (“LOOK”) condition. Similarly, the multivariate psychophysiological dissimilarity between each participant’s unique uninstructed and suppress conditions represents how much they naturally suppress in the uninstructed (“LOOK”) condition.

**Fig 5:**
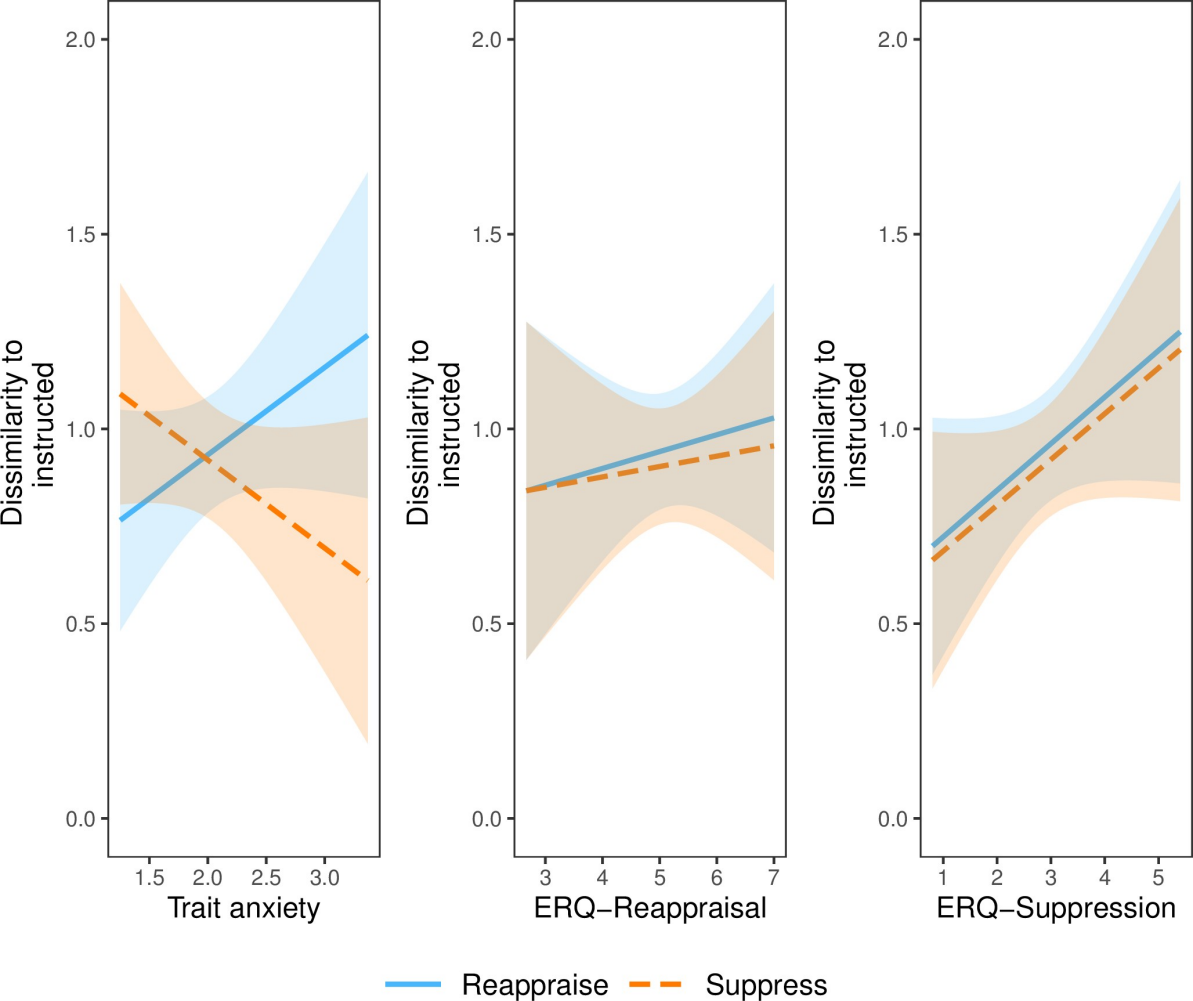
Individual differences in multivariate psychophysiological dissimilarity. Participants’ self-reported regulation tendency did not differentiate how they naturally regulated, but their trait anxiety did. Effects from Models 1, 2 and 3 on multivariate psychophysiological dissimilarity between instructed and uninstructed conditions. Left (Model 1): significant interaction between trait anxiety (STAI) and instructed condition. Middle (Model 2): non-significant interaction between ERQ-Reappraisal and instructed condition. Right (Model 3): non-significant interaction between ERQ-Suppression and instructed condition. Dissimilarity is measured with correlation distance (ranging from 0 to 2, with 0 representing perfect correlation, 1 representing no correlation, and 2 representing perfect anti-correlation). Smaller y-axis values represent greater similarity. ERQ scores are from the Emotion Regulation Questionnaire [36] and trait anxiety scores are from the State Trait Anxiety Inventory-trait Subscale [52].

We tested if participants’ self-reported regulation tendency and trait anxiety distinguished their psychophysiological profiles during instructed and uninstructed regulation. Specifically, we examined if participants’ self-reported regulation tendency and trait anxiety predicted if their psychophysiological profiles resembled a reappraisal profile or a suppression profile. To do this, we ran three linear mixed-effects regressions that allowed for random subject intercepts predicting psychophysiological dissimilarity between uninstructed and instructed regulation. Model 1 included instructed condition and STAI as regressors; Model 2 included instructed condition ERQ-Reappraisal as regressors; Model 3 included instructed condition and ERQ-Suppression as regressors. All 3 models predicted the same dependent variable—distance between uninstructed and instructed regulation conditions. To be clear, the effect of instructed condition (a categorical variable determining if the uninstructed psychophysiological profile was compared to either instructed reappraise or instructed suppress) was identical across all three models. All 3 models noted a significant effect of instructed condition (β = -.04, 95% CI [-.06, -.02], *p* < .001), simply demonstrating that there is a difference between the dissimilarity between uninstructed and reappraise versus uninstructed and suppress. Specifically, psychophysiological profiles are more similar, on average, between uninstructed and suppress than uninstructed and reappraise. Fig 5 illustrates effects from Models 1, 2 and 3.

Model 1 revealed that participants’ trait anxiety significantly distinguished if their uninstructed psychophysiological profiles resembled reappraisal versus suppression (β = -.45, 95% CI [-.05, -.04], *p* < .001). Specifically, trait anxiety interacted with instructed condition such that participants with higher anxiety showed uninstructed psychophysiological profiles resembling suppression, whereas participants with lower anxiety showed uninstructed psychophysiological profiles resembling reappraisal. There was no main effect of trait anxiety (β = .22, 95% CI [-.08, .52], *p* = .15).

Model 2 revealed that self-reported reappraisal tendency did not distinguish participants’ psychophysiological profiles. In addition to the aforementioned significant effect of instructed condition, the main effects of ERQ-Reappraisal (β = .04, 95% CI [-.12, .21], *p* = .61) and interaction between ERQ-Reappraisal and instructed regulation condition (β = -.02, 95% CI [-.04, .01], *p* = .23) were non-significant predictors of psychophysiological dissimilarity between uninstructed and instructed regulation. In other words, participants who reported frequently reappraising did not show a psychophysiological profile that resembled reappraisal.

Model 3 revealed that self-reported suppression tendency did not distinguish participants’ psychophysiological profiles. In addition to the aforementioned significant effect of instructed condition, the main effects of ERQ-Suppression (β = .12, 95% CI [-.02, .26], *p* = .11) and interaction between ERQ-Suppression and instructed regulation condition (β = -0, 95% CI [-.03, .02], *p* = .86) were non-significant predictors of psychophysiological dissimilarity between uninstructed and instructed regulation. In other words, participants who reported frequently suppressing did not show a psychophysiological profile that resembled suppression.

## Discussion

Emotion research has examined individual differences in affective experiences with self-report and psychophysiology. Most research relies on instructing individuals when and how to regulate their emotions, and self-report measures often intend to capture how individuals typically regulate outside the laboratory. But self-report measures of emotion and emotion regulation may not always cohere with psychophysiology. Research strives for ecological validity and hopes for alignment between how individuals report tending to regulate in the real world and how they regulate during an experiment. Moreover, how an individual performs during an emotion-regulation training is often assumed to indicate future performance inside and outside the laboratory. However, individuals may not always regulate similarly inside and outside the laboratory, but these relationships are seldom directly explored. To bridge this gap, we compared self-reported and psychophysiological measures of emotion and emotion regulation. To do this, we computed a multivariate psychophysiological measure of how much participants naturally reappraised and suppressed—free of instruction—then examined how closely this measure converged with self-report. Our results indicate that anxiety, but not regulation tendency, predicts how individuals regulate emotion in the laboratory. These findings suggest that how individuals report regulating in the real world does not map on to how they regulate in the laboratory.

Based on the logistic regression demonstrating the patterns of psychophysiological activity that differentiate reappraisal from suppression, individuals flattened their affect (according to EMG activity) and had higher skin conductance when suppressing compared to reappraising, which aligns to prior research [20–27, 31]. Moreover, participants who were the most anxious flattened their affect more and exhibited the highest levels of skin conductance activity. In line with a suppression profile, participants with lower anxiety did not exhibit increased skin conductance activity when naturally engaging with negative stimuli, yet were more emotionally expressive [15, 27]. Consistent with prior research, this suggests that trait anxiety influences not only how individuals freely engage with negative stimuli, but also how they respond to regulation instructions [13–17]. Surprisingly, reappraisal was associated with increased corrugator activity, which was inconsistent with our predictions. Similarly, reappraisal was associated with increased levator activity—but only for highly anxious individuals. This may suggest that although reappraisal may decrease the subjective experience of negative emotion, it may not always impact other channels of emotion, such as facial expression.

These findings are also consistent with previous research demonstrating a psychophysiological marker of emotion regulation. We collected EMG and EDA activity to measure psychophysiological profiles of emotion regulation. Although EMG is traditionally used to study emotional experiences and communication [20, 34, 60, 61], it can be used as a biomarker for emotion regulation as well [32, 62], as underlying emotional experiences and expressions change in response to the successful regulation of emotion. Based on these psychophysiological underpinnings of regulation, we examined the relationship between self-reported and psychophysiological measures of emotion and emotion regulation.

Participants’ self-reported regulation tendency did not significantly differentiate how they naturally regulated, *but their trait anxiety did*. As predicted, participants with *lower levels of anxiety* exhibited similar psychophysiological profiles when naturally regulating and following instructions to reappraise, suggesting they naturally reappraise more. Conversely, participants with *higher levels of anxiety* exhibited similar psychophysiological profiles when naturally regulating and following instructions to suppress, suggesting they naturally suppress more. However, the current study did not identify a relationship between psychophysiological profiles of regulation and self-reported regulation tendency.

Taken together, these results suggest that anxiety may be a better indicator than self-reported regulation tendency of how individuals regulate in the laboratory. Compared to subjective measures of emotion-regulation tendency, there is stronger coherence between subjective measures of anxiety and psychophysiological measures of emotion regulation. In line with prior research [13, 14], anxiety fundamentally shapes how individuals experience emotion. However, self-reported regulation style may not always capture how individuals regulate in the laboratory. This is critical because a) self-reported regulation tendency is often treated as the ground truth, b) coherence among various response systems is indicative of well-being and mental stability [33] and c) performance in an experiment is often interpreted as indicative of future behavior outside the laboratory. Measuring trait anxiety in conjunction with psychophysiology offers a new method for studying coherence between subjective and psychophysiological measures of emotion and emotion regulation.

This study is not without limitations. First, our psychophysiological measures investigated how facial expressions and arousal change as a function of regulating emotion. However, suppression may also involve verbal and behavioral changes [8], which we did not investigate. Similarly, suppression, by definition, involves altering motor movements, which influence skin conductance differently than reappraisal [27]. Therefore, it is difficult to explicate changes in skin conductance from suppressing versus simply the physical effort and attentional focus required to implement suppression. However, though arousal on its own is a non-specific measure not necessarily indicative of a specific type or valence of emotion, we consider changes in arousal in the context of changes in emotional expression and in response to engaging with stimuli shown to elicit negative emotion [20, 63]. Second, we measured how participants naturally engaged with negative emotion in an uninstructed (“LOOK”) condition. However, these trials were conducted after being trained on reappraisal and suppression and that prior training may have influenced responses to the uninstructed modulation of negative emotion condition. Though this is a common paradigm [7, 64] and blocks of trials were presented in a randomized order to reduce carryover effects, it may be unclear how much variability in psychophysiological profiles in the uninstructed condition was due to dispositional differences in regulation tendency versus priming from the training session. Future work should aim to collect uninstructed data prior to training in order to truly gauge how individuals naturally choose to regulate of their own volition.

Importantly, participants viewed negative stimuli and either naturally engaged, reappraised or suppressed. Pairwise-distances in three-dimensional space (corrugator, levator and skin conductance activity) were used to determine how similar participants’ natural (uninstructed) profiles were to their reappraisal and suppression profiles. Based on the focus of reappraisal and suppression in the current study, the analyses are limited to quantifying how much participants’ natural regulation profiles resembled reappraisal and suppression profiles. To align to the strategies measured with the ERQ and heavily associated with the STAI, the current study limited its focus to reappraisal and suppression. However, individuals may regulate in numerous ways [65]. The current study’s analyses therefore do not speak to how much participants’ natural regulation profiles do or do not resemble other regulation strategies. Moreover, some participants’ natural regulation profiles were not similar to either a reappraisal or suppression profile, as indicated by a correlation dissimilarity of 1, suggesting they may be regulating in different ways. Future work should include additional strategies to more comprehensively study variability in the uninstructed modulation of negative emotion.

Another question that warrants further research is how coherence between subjective and psychophysiological indices of regulation varies based on state versus trait experiences. The state-trait taxonomy has allowed research to emphasize differences between processes pertaining to transient versus stable responses. However, research has also demonstrated that this distinction is often arbitrary and lacking a clear boundary. For example, the classification of state versus trait is often attributable to no more than how experimental instructions are phrased [66, 67]. Specifically, though state measures of emotion and emotion regulation are tied to the specific environmental demands, trait measures, such as the ERQ and STAI-trait subscale used in the present study, are inextricably linked to states and amount to the summation of a series of states [66]. This study collected data about natural regulation tendency and anxiety with trait-based measures—and did not collect state or trial-by-trial data. Therefore, the current study explored how stable affective traits influence how individuals *tend to* experience and engage with negative emotion. Future research should explicate how stable affective tendencies differ from state measures in terms of subjective and psychophysiological coherence.

Importantly, this was an exploratory analysis aimed at quantifying spontaneous regulation of emotion. These data were collected as part of a different study (see [47]) and the sample size was idealized for a different empirical question. We hope these exploratory findings inspire future research to validate and extend what we know about dispositional emotion regulation in a larger sample collected for this empirical question.

### Future directions

These findings illuminate key differences in how anxiety influences how individuals respond naturally to negative stimuli and *respond to instructions to regulate*. Most therapeutic protocols and interventions for depression and anxiety are based on regulation training that is commonly applied to a wide audience [68]. This work indicates that instructed regulation may not be well captured by a one-size-fits-all model. These findings highlight the need for personalized treatment paradigms and introduce a potential platform for detecting individuals that may respond to different styles of emotion-regulation interventions. In addition, these findings suggest that how individuals report regulating in the real world does not map on to how they regulate in the laboratory [65]. Taken together, this underscores the importance of developing emotion-regulation interventions and paradigms that more closely align to and predict real-world outcomes.

These findings reveal a puzzle in emotion regulation—either a) individuals are not reporting their true regulation style or b) how individuals regulate in the real world is not captured by laboratory experiments. Individuals may find it challenging to reflect on and characterize how they regulate when they are not actively regulating in that moment. Moreover, individuals may not be able to sum up their regulation style in a single measure—how they choose to regulate may greatly vary based on the situation and their personal goals. Regulation may be too contextual and idiosyncratic to varying goals and circumstances to distill into a single dispositional tendency [17, 69], highlighting the importance of conducting research outside the laboratory [70]. However, anxiety may be a more stable trait that better predicts how individuals regulate. Anxiety may be the more fundamental disposition, which can be accurately captured by self-report, and informs how individuals regulate [71]. Future research should explore this puzzle further to understand the disconnect between self-report and laboratory measures of emotion.
